# Propofol Directly Increases Tau Phosphorylation

**DOI:** 10.1371/journal.pone.0016648

**Published:** 2011-01-31

**Authors:** Robert A. Whittington, László Virág, François Marcouiller, Marie-Amélie Papon, Noura B. El. Khoury, Carl Julien, Françoise Morin, Charles W. Emala, Emmanuel Planel

**Affiliations:** 1 Department of Anesthesiology, College of Physicians and Surgeons, Columbia University, New York, New York, United States of America; 2 Centre Hospitalier de l'Université Laval, Neurosciences, Québec, Canada; New York State Institute for Basic Research, United States of America

## Abstract

In Alzheimer's disease (AD) and other tauopathies, the microtubule-associated protein tau can undergo aberrant hyperphosphorylation potentially leading to the development of neurofibrillary pathology. Anesthetics have been previously shown to induce tau hyperphosphorylation through a mechanism involving hypothermia-induced inhibition of protein phosphatase 2A (PP2A) activity. However, the effects of propofol, a common clinically used intravenous anesthetic, on tau phosphorylation under normothermic conditions are unknown. We investigated the effects of a general anesthetic dose of propofol on levels of phosphorylated tau in the mouse hippocampus and cortex under normothermic conditions. Thirty min following the administration of propofol 250 mg/kg i.p., significant increases in tau phosphorylation were observed at the AT8, CP13, and PHF-1 phosphoepitopes in the hippocampus, as well as at AT8, PHF-1, MC6, pS262, and pS422 epitopes in the cortex. However, we did not detect somatodendritic relocalization of tau. In both brain regions, tau hyperphosphorylation persisted at the AT8 epitope 2 h following propofol, although the sedative effects of the drug were no longer evident at this time point. By 6 h following propofol, levels of phosphorylated tau at AT8 returned to control levels. An initial decrease in the activity and expression of PP2A were observed, suggesting that PP2A inhibition is at least partly responsible for the hyperphosphorylation of tau at multiple sites following 30 min of propofol exposure. We also examined tau phosphorylation in SH-SY5Y cells transfected to overexpress human tau. A 1 h exposure to a clinically relevant concentration of propofol *in vitro* was also associated with tau hyperphosphorylation. These findings suggest that propofol increases tau phosphorylation both *in vivo* and *in vitro* under normothermic conditions, and further studies are warranted to determine the impact of this anesthetic on the acceleration of neurofibrillary pathology.

## Introduction

Alzheimer's disease is the leading cause of dementia and as of 2006, the worldwide prevalence of the disease was 26.6 million [1]. Alzheimer's disease is histopathologically characterized by the presence of extracellular senile plaques comprised primarily of aggregated β-amyloid peptides [2], as well as intraneuronal neurofibrillary tangles (NFTs) that are composed of insoluble aggregates of hyperphosphorylated tau protein [3], [4]. Tau protein is a microtubule-associated protein abundantly found in neuronal axons. In AD and other neurodegenerative tauopathies, tau can be abnormally hyperphosphorylated, leading to its self-assembly into straight or paired helical filaments, the major components of NFTs [5], [6].

The etiology of Alzheimer's disease is probably multifactorial, and there is growing interest in the potential relationship between anesthesia and the onset and progression of Alzheimer's disease [7], [8], [9], [10]. Many pre-clinical studies have demonstrated that anesthetics can enhance β-amyloid toxicity and aggregation both *in vitro* and *in vivo* [11], [12], [13], [14]. In terms of tau protein, we demonstrated that both inhalational and intravenous anesthetics produce tau hyperphosphorylation through a mechanism involving the inhibition of protein phosphatase 2A (PP2A) activity by anesthesia-induced hypothermia [15]. We have also shown that anesthesia can affect tau functional dynamics; in a transgenic mouse model expressing all 6 isoforms of normal human tau, pentobarbital-induced hypothermia hyperphosphorylates tau, thus impairing its ability to bind and assemble microtubules (MTs) [16]. Moreover, in a transgenic mouse model of tau pathology, we observed that isoflurane-induced hypothermia accelerates neurofibrillary pathology [17].

It has been recently demonstrated that anesthetics can also produce tau hyperphosphorylation in the absence of hypothermia [18]. However, this study focused on the effects of ether and sodium pentobarbital, anesthetics that are rarely used in current clinical anesthesia practice. Propofol (2,6 diisopropylphenol) is an intravenous sedative-hypnotic, commonly used as an anesthetic for procedures requiring general anesthesia and conscious sedation as well as for prolonged sedation in intensive care units [19]. Given the recent findings by Run *et al.* [18], we investigated whether a general anesthetic dose of propofol would increase the phosphorylation of tau in the absence of hypothermia. Hence, the purpose of this study was to examine the direct impact of propofol on tau phosphorylation under normothermic conditions *in vivo,* as this would more closely mimic clinical anesthetic exposure. We observed that propofol leads to tau hyperphosphorylation under normothermic conditions both in mice and in human neuroblastoma cells.

## Results

### Propofol Increases Tau Phosphorylation Under Hypothermic and Normothermic Conditions in the Mouse Hippocampus

**Figure 1:**
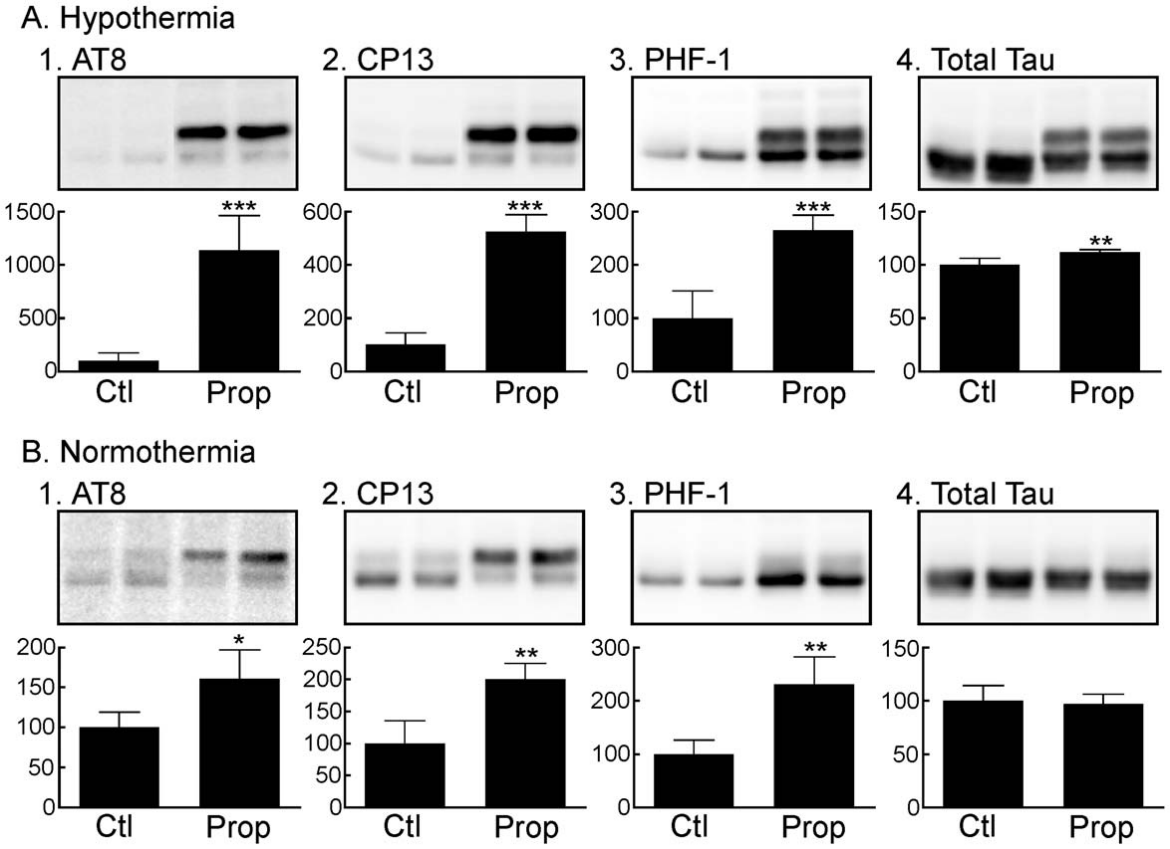
Tau phosphorylation in mouse hippocampal tissue 30 min following the administration of propofol under hypothermic (*A*) or normothermic (*B*) conditions. Hippocampal protein extracts were separated by SDS-PAGE and levels of tau phosphorylation were determined using antibodies directed at the AT8 (Ser^202^/Thr^205^;*A1,B1*), CP13 (Ser^202^;*A2,B2*), and PHF-1 (Ser^396^/Ser^404^;*A3,B3*) phosphoepitopes, or total tau (*A4, B4*). Relative immunoreactive band intensities are expressed as a percent of control (Ctl; intralipid) and are displayed for each phosphoepitope and total tau. For each condition, 2 representative data are displayed with Ctl (n = 4), and Prop (n = 5). Data are expressed as mean ± SD. *** denotes *P*<0.001, ** denotes *P*<0.01, and * denotes *P*<0.05 vs. Ctl with unpaired *t*-test.

We first wanted to confirm that propofol would induce hypothermia-mediated tau hyperphosphorylation, as it has not been reported previously. Thirty min following propofol administration, significant hypothermia occurred in the group of mice whose temperature was not controlled during anesthesia (Hypothermia, n = 5). The mouse rectal temperature decreased to 30.9±1.0°C in the hypothermia group versus 37.5±0.4 and 37.5±0.6°C in the control (n = 4) and temperature controlled (Normothermia, n = 5) groups, respectively (*P*<0.001 vs. control). As expected, given that anesthesia-induced hypothermia inhibits the activity of PP2A [15], propofol-induced hypothermia produced a significant increase in hippocampal tau phosphorylation at the AT8 (1135±325%), PHF-1 (265±28%), and CP13 (525±63%) phosphoepitopes (Fig.1A1, A2, A3), when compared to the control group, and there was a slight increase (112±2%) in total tau in the hypothermic group (Fig. 1A4). Nevertheless, significant tau hyperphosphorylation at AT8 (161±36%), PHF-1 (231±5 1%), and CP13 (200±25%) was also observed in the hippocampal tissue of the normothermic group (Fig.1B1, B2, B3). Overall, our data indicate propofol can induce tau hyperphosphorylation by inducing hypothermia, but can also do so in the absence of hypothermia, albeit to a lesser extent.

### Hyperphosphorylated Tau does not Relocalize in the Somato-Dendritic Compartment

**Figure 2:**
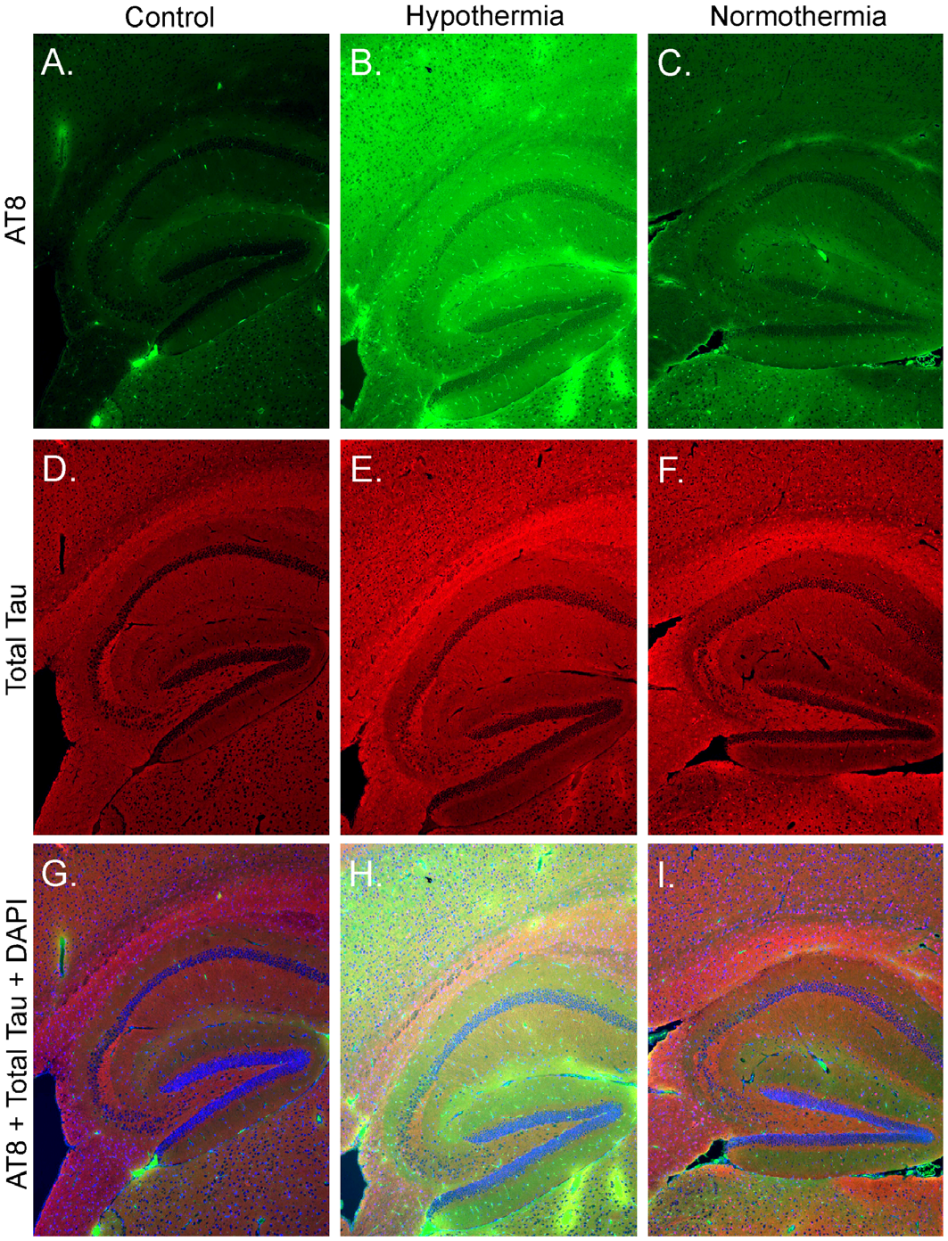
Regional anatomical localization of tau phosphorylation following 30 min of anesthesia with propofol. Fluorescence photomicrographs of hippocampal sagittal sections are shown with AT8 (Green, *A,B,C*), Total Tau (Red, *D,E,F*), or merged with DAPI (Green-Red-Blue, *G,H,I*), for the following conditions: Control (Intralipid, *A,D,G*), Hypothermia (*B,E,H*), and Normothermia (*C,F,G*). All images were taken at 5x magnification.

In the normal adult brain, tau is an axonal protein, with a typical pattern of staining in the neuropil and axonal tracks, but not in the somatodendritic compartment. In AD, tau-positive dystrophic axons are widespread, and the earliest detectable hyperphosphorylated tau is preferentially localized in neurites of vulnerable neurons before extending to the soma [20]. We thus examined the gross anatomical pattern of tau phosphorylation, to see whether propofol-induced tau hyperphosphorylation would induce a somadendritic relocalization of tau, one of the early signs of tau pathology. There was a robust hyperphosphorylation of tau at the AT8 epitope 30 min after propofol injection under hypothermic conditions (Fig. 2B), and to a much lesser extent 30 min after propofol treatment under normothermic conditions (Fig. 2C). However, there was no observable change in total or phospho-tau cellular localization, as revealed by AT8, Total Tau, and DAPI staining patterns. These results confirm our Western blot data, which revealed large changes in tau phosphorylation at the AT8 epitope (Fig. 1A) in response to propofol-induced hypothermia, but smaller changes in phosphorylation when the animals were kept normothermic (Fig. 1B), and reveal that short exposure to propofol, in either normothermic or hypothermic conditions does not induce pathological somato-dendritic relocalization of tau.

### Tau Hyperphosphorylation Under Normothermic Conditions Persists at the AT8 Phosphoepitope in the Mouse Hippocampus Following Recovery From Anesthesia

**Figure 3:**
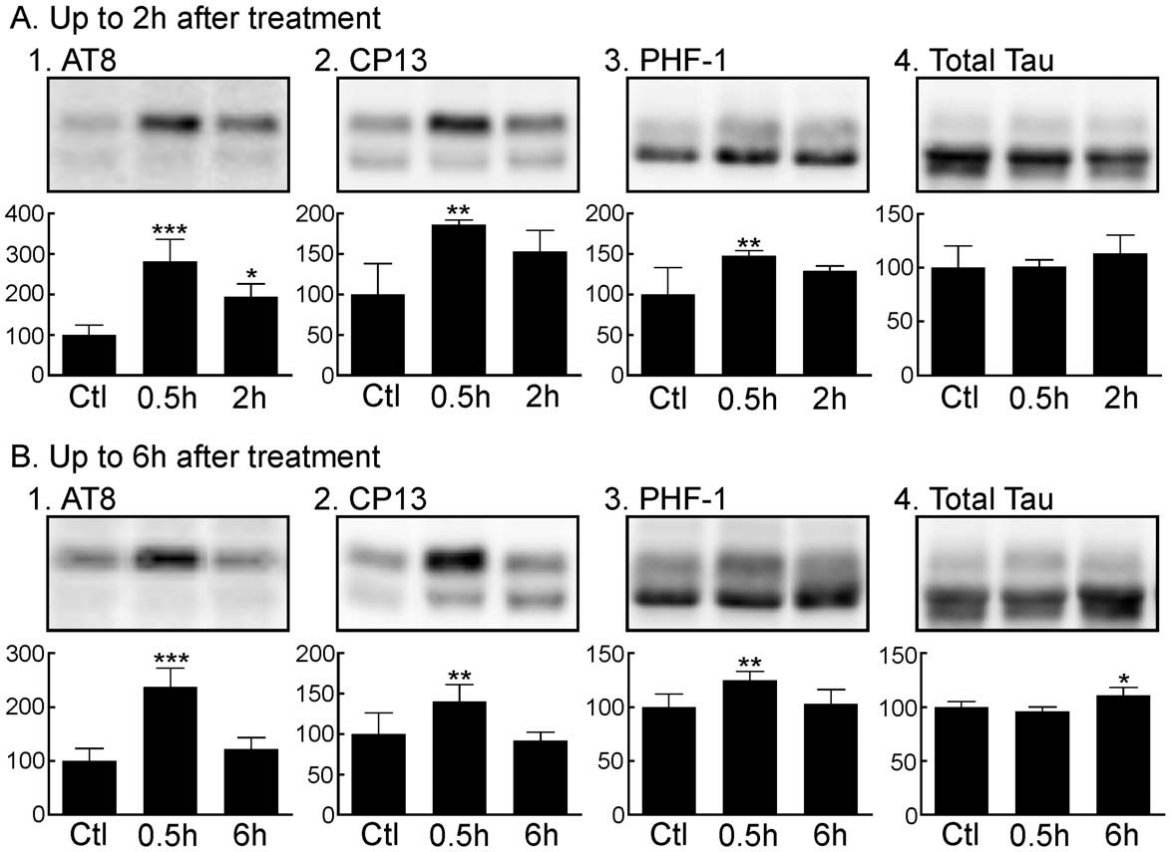
Tau phosphorylation in mouse hippocampal tissue 0.5 h and 2 h (*A*), or 0.5 h and 6 h (*B*) following the administration of propofol under normothermic conditions. Hippocampal protein extracts were separated by SDS-PAGE and levels of tau phosphorylation were determined using antibodies directed at the AT8 (*A1, B1*), PHF-1 (A2, *B2*), and CP13 (*A3, B3*) phosphoepitopes, or Total Tau (*A4, B4*). Relative immunoreactive band intensities are expressed as a percent of control (Ctl; intralipid) and are displayed for each phosphoepitope and total tau. For each condition, 1 representative datum is displayed with Ctl (n = 4), 0.5 h (n = 5), 2 h (n = 5), or 6 h (n = 5). Data are expressed as mean ± SD. *, ** and *** denote *P*<0.05, *P*<0.01 and *P*<0.001 vs. ctl, respectively; ANOVA with Newman-Keuls *post hoc* test.

We next determined whether the increase in hippocampal tau phosphorylation under normothermic conditions persists following recovery from propofol anesthesia. Thirty minutes following the administration of propofol, hippocampal tau phosphorylation at the AT8 epitope significantly increased to 282±54% of control (Fig. 3A1), confirming our previous results. However, in the group sacrificed 2 h following propofol, despite the recovery of the righting reflex in all of these mice, hippocampal tau hyperphosphorylation persisted at the AT8 epitope and was 195±31% of control (Fig. 3A1). Interestingly, there were significant increases in hippocampal tau phosphorylated at the PHF-1 (148±6%) and CP13 (153±26%) phosphoepitopes 30 min following propofol (Fig. 3A2, A3); however, 2 h following anesthesia PHF-1 and CP13 phosphorylation was not significantly increased. Moreover, the increase in hippocampal tau phosphorylation at 30 min was greatest at AT8 when compared to the increases observed at PHF-1 and CP13. There were no observed changes in hippocampal total tau levels throughout the study (Fig. 3A4). Normothermia was maintained and rectal temperatures were similar in all 3 groups: Control  = 37.3±0.2°C, Prop 30 min  = 36.8±0.2°C, and Prop 2 h  = 37.1±0.4°C.

Because of this persistence of tau hyperphosphorylation at the AT8 phosphoepitope, we performed a subsequent set of experiments in C57BL/6 mice to determine whether tau phosphorylation at this specific phosphoepitope returns to control levels 6 h following propofol administration. Normothermia was maintained throughout and rectal temperatures were similar in all 3 groups: Control  = 37.1±0.3°C, Prop 0.5 h  = 36.9±0.2°C, and Prop 6 h  = 37.0±0.3°C. As previously observed, 30 min (Prop 0.5 h, n = 5) following propofol, tau phosphorylation at AT8, PHF-1, and CP13 significantly increased compared to control (Ctrl, n = 4); however, by 6 h (Prop 6 h, n = 5) following propofol, tau phosphorylation had returned to control levels at all 3 phosphoepitopes (Fig. 3B1, B2, B3). There was a slight increase of total tau after 6 h (111±7%). Of note, in this group of mice, the increase in tau phosphorylation at AT8 was again greater than that observed at PHF-1 and CP13.

Hence, under normothermic conditions, propofol produces an immediate increase in tau phosphorylation at the AT8, PHF-1, and CP13 phosphoepitopes; however, at the AT8 phosphoepitope tau hyperphosphorylation persists 2 h following anesthesia but recovers 6 h following propofol treatment. Furthermore, the greater degree and longer duration of hyperphosphorylation at AT8, when compared to PHF-1 and CP13, suggests that this phosphoepitope is more susceptible to the hyperphosphorylation effects of propofol.

### Propofol Induces Transient Tau Hyperphosphorylation in the Mouse Cortex Under Normothermic Conditions

To determine whether regional brain differences in tau phosphorylation exist, we also examined levels of tau phosphorylation at the AT8, PHF-1, pS262, MC6 and pS422 phosphoepitopes in cortical tissue harvested 30 min (0.5 h, n = 6) or 2 h (2 h, n = 7) following the administration of propofol or intralipid (Ctrl, n = 6). In the cortex, tau phosphorylation at the AT8 phosphoepitope increased to 154±28% of control 30 min and to 144±29% of control 2 h following propofol administration (Fig. 4.1), confirming the results from the hippocampal tissues. Moreover, a significant increase in tau phosphorylation was observed in the cortex at the PHF-1 (141±30%), pS262 (225±112%), MC6 (143±42%), and pS422 (130±21%) epitopes 30 min but not 2 h after propofol treatment (Fig. 4.2 to 4.5). Propofol produced no significant change in total tau (Fig. 4.6) or β-tubulin (data not shown) in the cortex. Again, these data demonstrate that tau hyperphosphorylation following propofol is also observed in the cortex, and that the AT8 epitope is especially susceptible to propofol-induced hyperphosphorylation.

**Figure 4:**
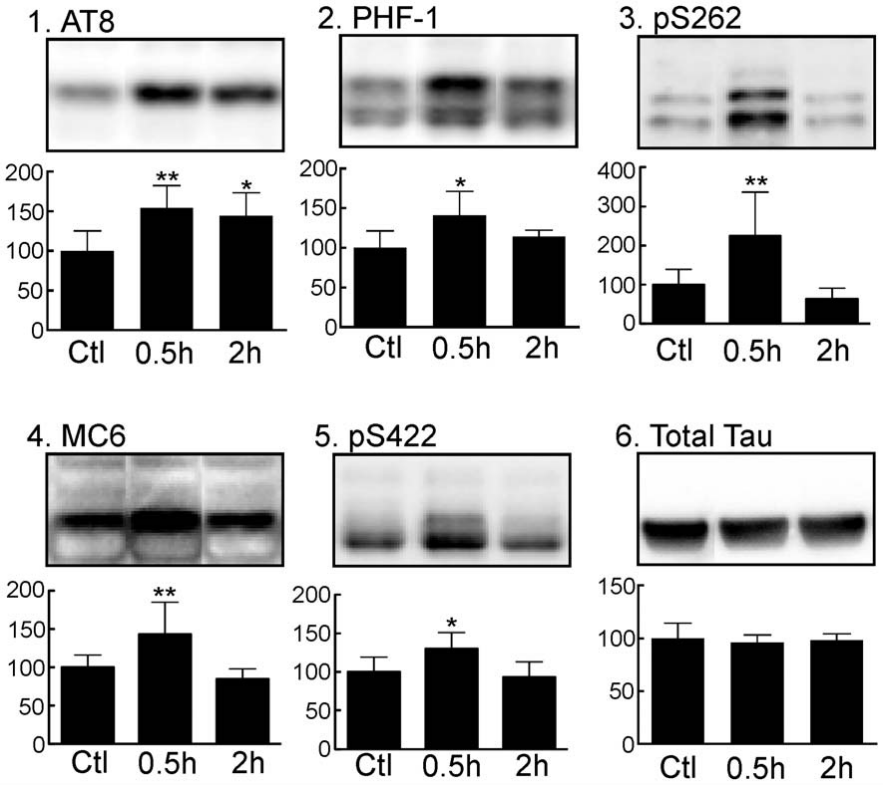
Tau phosphorylation in mouse cortical tissue 30 min and 2 h following the administration of propofol under normothermic conditions. Cortical protein extracts were separated by SDS-PAGE and levels of tau phosphorylation were determined using antibodies directed at the AT8 (*1*), PHF-1 (*2*), pS262 (*3*), MC6 (*4*), and pS422 (*5*) phophoepitopes, or Total Tau (*6*). Relative immunoreactive band intensities are expressed as a percent of control (Ctl; intralipid) and are displayed for each phosphoepitope and total tau. For each condition, 1 representative datum is displayed with Ctl (n = 6), 0.5 h (n = 6), and 2 h (n = 7). Data are expressed as mean ± SD. *, and ** and denote *P*<0.05, and *P*<0.01 vs. Ctl, respectively; ANOVA with Newman-Keuls *post hoc* test.

### Impact of Propofol on Tau Kinases and Phosphatases

**Figure 5:**
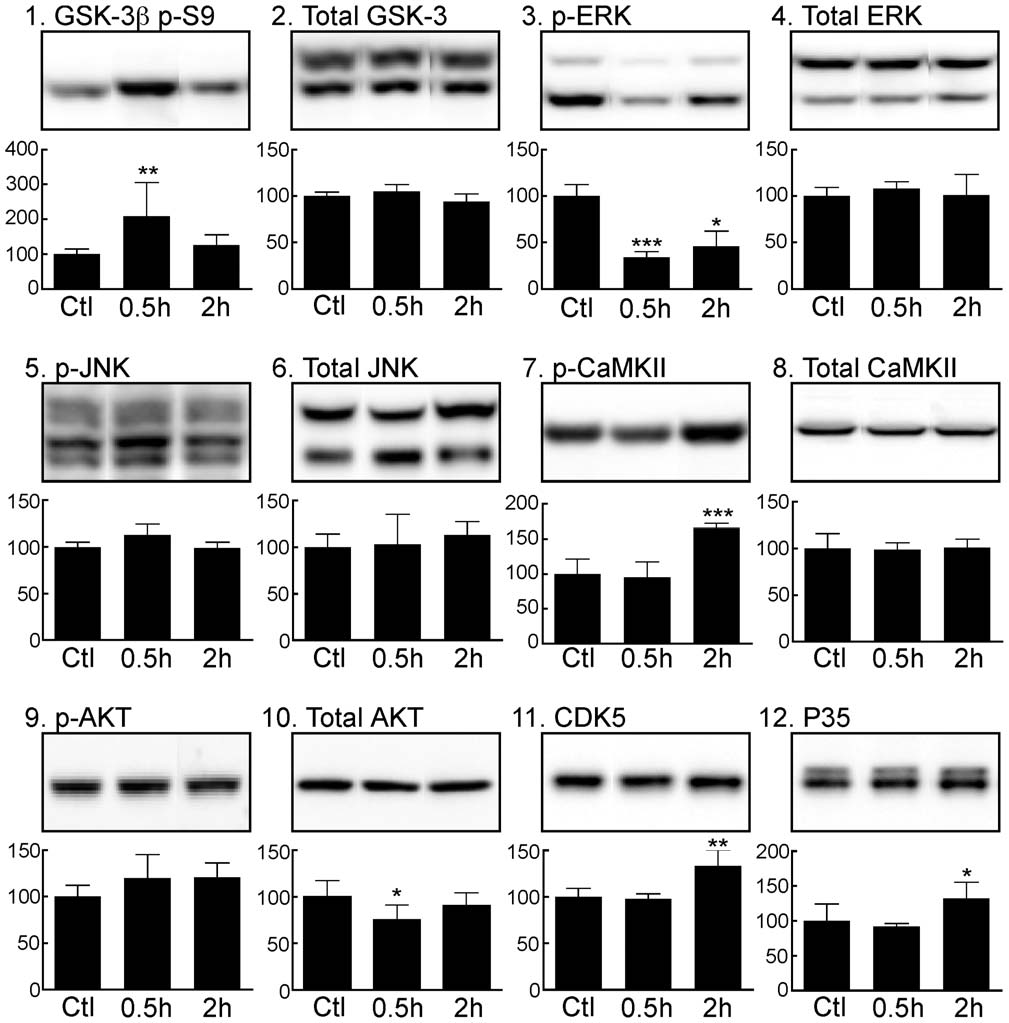
Effect of propofol on tau kinases in mouse cortical tissue 0.5 h and 2 h following the administration of propofol under normothermic conditions. Cortical protein extracts were separated by SDS-PAGE and levels of kinases were determined using antibodies directed at activated or total kinases as follow: (*1*) GSK-3β phospho-S9, (*2*) total GSK-3 (α and β), (*3*) phospho-ERK, (*4*) total ERK, (*5*) phospho-JNK, (*6*) total JNK, (*7*) phospho-CaMKII, (*8*) total CaMKII, (*9*) phospho-AKT, (*10*) total AKT, (*11*) CDK5, and (*12*) P35. Relative immunoreactive band intensities are expressed as a percent of control (Ctl; intralipid) and are displayed for each epitope. For each condition, 1 representative datum is displayed with Ctl (n = 6), 0.5 h (n = 6), and 2 h (n = 7). Data are expressed as mean ± SD. *, ** and *** denote *P*<0.05, *P*<0.01 and *P*<0.001 vs. ctl, respectively; ANOVA with Newman-Keuls *post hoc* test.

As our results demonstrate that tau hyperphosphorylation following propofol anesthesia occurs under normothermic conditions, we next examined the activation state of specific tau kinases as well as the protein expression levels of phosphatases commonly involved in the regulation of tau phoshorylation. Among the kinases capable of phosphorylating tau, glycogen synthase kinase-3β (GSK-3β), cyclin-dependent kinase 5 (cdk5) and p35 (a specific activator of cdk5), mitogen-activated protein kinase/extracellular signal-regulated kinase (MAPK/ERK), and c-Jun N-terminal kinase (JNK) are considered to be major physiological and pathological tau kinases [21], [22], [23]. AKT/PKB (Protein Kinase B) can enhance tau phosphorylation directly [24] or indirectly by modulating the inhibitory phosphorylation of GSK-3β at Ser^9^ [25]. CaMKII is also thought to have a major role in regulating the phosphorylation of tau at epitopes that modulate the binding of tau to microtubules [26]. We explored the activation patterns of these six kinases with specific antibodies in the same cortical tissues obtained from mice that received propofol 250 mg/kg i.p. and were subsequently sacrificed 30 min or 2 h following drug administration. Thirty minutes following propofol there was significant increase in p-GSK-3β (Ser 9) levels (Fig. 5.1), a significant decrease in p-ERK (Fig. 5.3), and a slight decrease in total AKT levels (Fig. 5.10), but all the other kinases examined did not change (Fig. 5). Two hours following propofol the decrease in p-ERK persisted while p-CaMKII was significantly increased (Fig. 5.7); there were also significant increases in CDK5 and P35 levels (Fig. 5.11 and 5.12). However, there was no correlative kinase activation that could explain the tau hyperphosphorylation observed 30 min after treatment.

We therefore examined next the levels of tau protein phosphatases (PP) catalytic subunits 30 min and 2 h after propofol administration. Tau can be dephosphorylated by PP1, PP2A, and PP2B, but PP2A shows a much stronger capacity to dephosphorylate tau [27], [28]. The only detectable change was a significant decrease in PP2A-C 30 min after treatment (Fig. 6.3; 76±2%), suggesting a decrease in PP2A activity, which was confirmed with an immunoprecipitation assay (Fig. 6.4; 77±9%).

**Figure 6:**
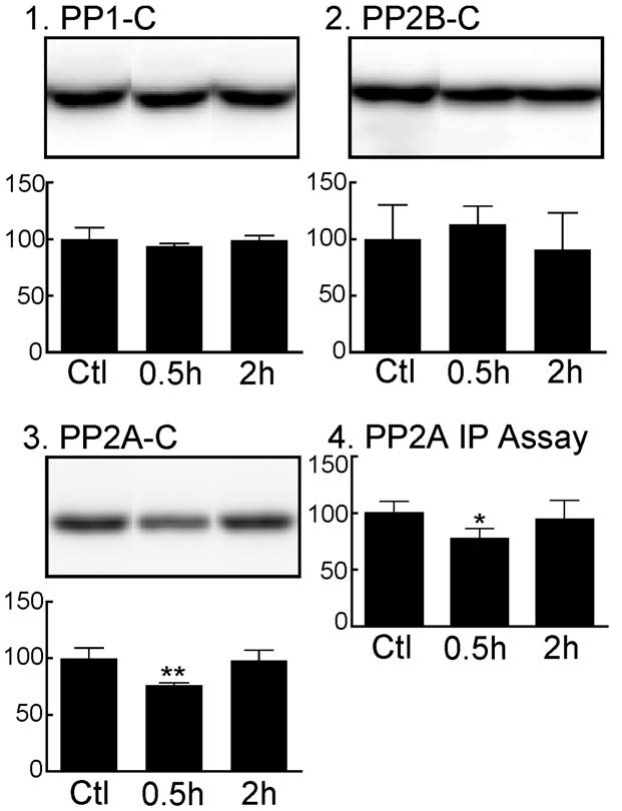
Effect of propofol on tau phosphatases in mouse cortical tissue 0.5 h and 2 h following the administration of propofol under normothermic conditions. Cortical protein extracts were separated by SDS-PAGE and levels of phosphatases were determined using antibodies directed at the following proteins: (*1*) PP1 catalytic subunit, (*2*) PP2B catalytic subunit, and (*3*) PP2A catalytic subunit. Relative immunoreactive band intensities are expressed as a percent of control (Ctl; intralipid) and are displayed for each epitope. For each condition, 1 representative datum is displayed. (*4*) PP2A activity was measured with the PP2A Immunoprecipitation Phosphatase BioAssay Kit from US Biological and values expressed as percentage of control. All data are expressed as mean ± SD. * and ** denote *P*<0.05 and *P*<0.01 vs. ctl, respectively; Ctl (n = 6), 0.5 h (n = 6), and 2 h (n = 7); ANOVA with Newman-Keuls *post hoc* test.

### Propofol Induces Transient Tau Hyperphosphorylation in SH-SY5Y cells

**Figure 7:**
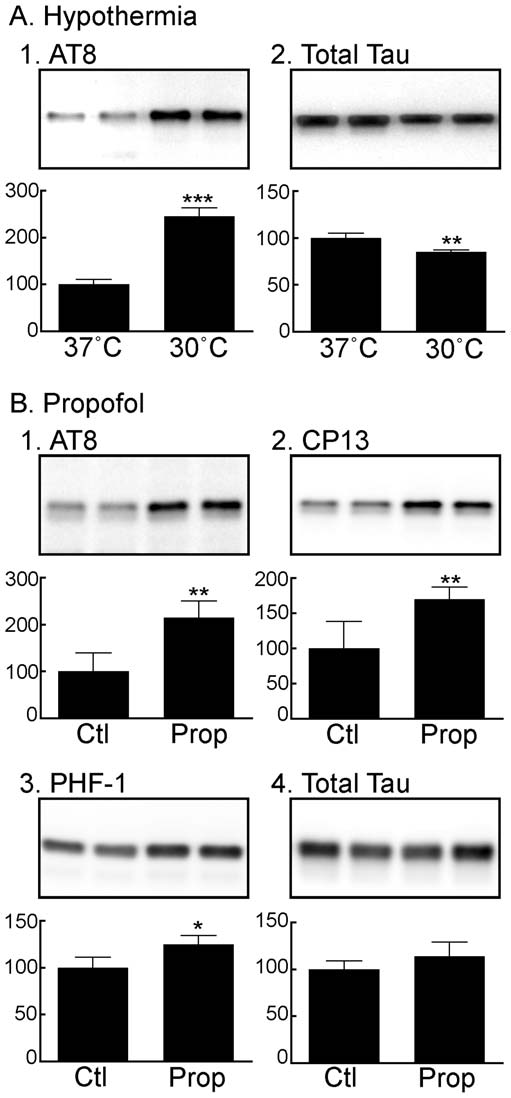
Effect of propofol on tau phosphorylation in Tau-SH-SY5Y cells. Cells were harvested after 1 h incubation at 37°C (n = 4) or 30°C (n = 3) in the absence of propofol (*A*), or following 1 h exposure to 10% intralipid in DMEM (Ctl, n = 3) or propofol (Prop, n = 5) at 3 µg/ml (16.8 µM), both at 37°C (*B*). Cell lysate protein extracts were separated by SDS-PAGE and the level of tau phosphorylation was determined using antibodies directed at the AT8 (*A1, B1*), CP13 (*B2*), or PHF-1 (*B3*) phosphoepitopes, or total tau (*A2, B4*). Relative immunoreactive band intensities are expressed as a percent of control and are displayed for each phosphoepitope and total tau. Tau phosphoepitopes are normalized on total tau. For each condition, 2 representative data are displayed. Data are expressed as mean ± SD, with *, **, and *** denoting *P*<0.05, *P*<0.01 and *P*<0.001 vs. ctl, respectively with unpaired *t*-test.

We next determined the effect of propofol on tau phosphorylation in vitro using SH-SY5Y human neuroblastoma cells stably transfected to overexpress human tau (Tau-SH-SY5Y cells). These studies were performed to determine whether propofol-induced tau hyperphosphorylation is the result of a direct pharmacologic effect and not the consequence of an anesthetic-induced physiologic change. First, as a positive control, we examined the degree of tau phosphorylation in the cells at the AT8 epitope following 1 h incubation at 37°C (control, n = 4) or 30°C (n = 3). Following a 1 h incubation of the Tau-SH-SY5Y cells at 30°C, tau phosphorylation increased to 245±18% of the control (37°C) group (Fig. 7A1), confirming that these transfected cells respond appropriately to hypothermia, an environmental condition known to increase tau phosphorylation. Total tau was slightly decreased (Fig. 7A2; 85±2%). In the propofol experiments, when compared to control (n = 3), 1 h following exposure to propofol 3 ug/ml (n = 5), a physiologic dose of propofol, tau phosphorylation at AT8 increased significantly in the Tau-SH-SY5Y cells to 215±35% of control (Fig. 7B1). Significant increases in phosphorylated tau at CP13 (170±17%; Fig. 7B2) and PHF-1 (125±9%; Fig. 7B3) were also observed with the 3 ug/ml concentration. No significant change in total tau (Fig. 7B4) or β-actin (data not shown) were observed among the 3 treatment groups. These results demonstrate that propofol can induce direct tau hyperphosphorylation in neuronal cells, and suggest that, as propofol can cross the blood-brain barrier [29], the outcome observed in mice is probably due to a direct pharmacologic effect on neurons.

## Discussion

The findings of the present study are novel and demonstrate that a common clinically used intravenous anesthetic, propofol, can increase tau phosphorylation in the mouse hippocampus and cortex, not only as a consequence of anesthesia-induced hypothermia, but also due to a direct effect of the drug itself. Furthermore, in the hippocampus, tau hyperphosphorylation at the AT8 phosphoepitope persists for at least 2 h following propofol administration before returning to control levels 6 h later, thus demonstrating that direct tau hyperphosphorylation by propofol can persist even following recovery from the sedative-hypnotic effects of the drug. The observation that normothermic hyperphosphorylation following anesthesia occurred within a clinically relevant time frame, 30 min and 2 h following exposure, is also novel.

We previously observed that the hyperphosphorylation of tau following intravenous and inhalational anesthetics primarily involves the inhibition of PP2A by anesthetic-induced hypothermia [15]. Our data clearly supports this previous finding as propofol-induced hypothermia produced tau hyperphosphorylation that was significantly greater than that observed under normothermic conditions. However, in contrast to our previous study, we observed a direct effect of an intravenous anesthetic on tau phosphorylation in the absence of hypothermia. One possible reason for this observed difference includes the fact that in the current study, we examined propofol, a phenol-based derivative that is structurally and chemically unrelated to the intravenous anesthetics we previously examined, namely pentobarbital and choral hydrate. Furthermore, in the aforementioned study, the normothermic animals were allowed to initially become hypothermic followed by a period of “rescue normothermia”. However, in the present study, normothermia was maintained throughout the administration of anesthesia, thus avoiding any temperature-related changes in tau-related kinase or phosphatase activity. Temperature fluctuations can affect the relative balance of tau-related kinase and phosphatase activity [30]; hence, the maintenance of normothermia throughout the study virtually eliminated the possibility that hypothermia-induced inhibition or activation of these enzymes would be a factor during the determination of the direct effects of propofol on tau phosphorylation.

More recent studies have observed that either ether or sodium pentobarbital anesthesia can directly lead to hyperphosphorylated tau both 30 sec and 5 min following treatment, as no evidence of hypothermia was observed by these investigators at either of these time points [18]. The present study expands on these findings by demonstrating the effects of a general anesthetic dose of a more clinically-utilized anesthetic at clinically relevant time intervals: 30 min and 2 h following anesthesia. Similar to Run *et al.,* we observed that the degree of hyperphosphorylation observed following anesthesia varies as a function of the specific phosphoepitope. Specifically, we observed the greatest increase in tau phosphorylation following 30 min of propofol treatment at the AT8 phosphoepitope, and to a lesser extent at CP13, PHF-1, pS262, MC6, and pS422, in the hippocampus or the cortex. Furthermore, the persistence of tau hyperphosphorylation at AT8 (pSer^202^/pThr^205^) after 2 h in both brain regions, also suggests that propofol has a greater capacity to produce hyperphosphorylation at certain phosphoepitopes.

As tau phosphorylation is regulated by the activity of several protein kinases [31], [32], [33] and protein phosphatases [34], [35], [36], we examined the expression and/or activation of several of these enzymes. Thirty min after propofol treatment, there was no increase in kinases activation, but the decrease in PP2A-C expression and PP2A activity, along with the increase of phospho-GSK-3β (Ser9) levels strongly suggests that propofol may be producing its effect on tau phosphorylation (at AT8, CP13, MC6, PHF-1, pS262, and pS422) not primarily by the activation of kinases, but rather through an inhibition of PP2A activity. Indeed, increased levels phospho-tau at all epitopes examined, along with increased phospho-GSK-3β (Ser9) have been consistently observed during inhibition of phosphatases, either by okadaic acid in neuronal cultures [36] or by hypothermia in mice [15], [30]. On the other hand, a significant decrease in p-ERK, and increases in levels of p-CaMKII, cdk5 and its activator p35 were also observed at 2 h. Among the phospho-tau epitopes examined here, ERK is known to phosphorylate AT8, CP13, MC6, PHF-1, and pS422; cdk5 can phosphorylate AT8, CP13, MC6, and PHF-1; CaMKII is more specific to pS262 (for a table recapitulating the kinases and their *in vitro* target epitopes on tau, see [22]). Thus, the increase in CaMKII activation was not reflected by an increase in pS262 levels, and p-ERK was diminished, which suggest that they are not directly implicated in the persistent phosphorylation of AT8 at 2 h. It should be noted that it is not the first time that we observe increased CaMKII activation not correlating with increased tau phosphorylation *in vivo*; for example, 10 days of diabetes induced by streptozotocin resulted in more than 1000% increase in p-CaMKII but no detectable phosphorylation at either pS262 or pS356, two sites demonstrated to be phosphorylated by CaMKII *in vitro* [48]. The increase in cdk5 and p35 might have explained the persistent phosphorylation at AT8 at 2 h, but other epitopes known to be phosphorylated by cdk5, such as MC6, CP13 and PHF-1 were not increased, which suggest that cdk5 is not directly responsible for the persistent AT8 phosphorylation. Overall, our results suggest that the mechanism of tau phosphorylation after propofol treatment is biphasic, with an inhibition of PP2A resulting in tau hyperphosphorylation at multiple epitopes after 30 min, followed by the persistence of AT8 phosphorylation at 2 h. This persistence might be due to residual phosphorylation or the activation of an unknown kinase specific for the AT8 site.

Although it would be premature to extrapolate these preclinical findings to the clinical sphere at this juncture, the observation that propofol has direct effects on tau phosphorylation should not be underestimated. This is especially true given the fact that unlike anesthetics previously studied in the context of their capacity to produce tau hyperphosphorylation such as isoflurane, ether, choral hydrate, and sodium pentobarbital [15], [17], [18], propofol is an anesthetic that is administered not solely intraoperatively for a few hours, but also for days as the drug is commonly used for post-operative sedation in critical care settings such as intensive care units. Indeed, Wunsch et al. recently demonstrated that propofol is currently the most common form of intravenous anesthetic utilized for sedation purposes in U.S. intensive care units [19]. Hence, our current finding of propofol-induced tau hyperphosphorylation and its persistence at select phosphoepitopes following a short period of general anesthesia, makes it plausible that this state of abnormal phosphorylation may also persist in the setting of prolonged administration. Given that post-operative cognitive impairment is commonly observed following intensive care [37], the observation that an anesthetic commonly used for ICU sedation is associated with a direct increase in tau phosphorylation suggests that the contribution of this hyperphosphorylation to the development of tau neurofibrillary pathology and neurocognitive impairment warrants further investigation.

## Materials and Methods

### Animals

The experimental protocol was approved by the Columbia University Animal Care and Use Committee and, in accordance with National Institutes of Health (NIH) guidelines, adequate measures were taken to minimize pain and discomfort. Seven to nine-week-old C57BL/6 male mice were purchased from a commercial vendor (Taconic, Germantown, NY) and utilized in this study. The mice were housed in a temperature-controlled room at 22°C and were kept on a 12 h/12 h light/dark cycle. All animals had access to food and water *ad libitum*, and they underwent an acclimatization period for a minimum of 24 h before being used in the experiments.

### Anesthesia exposure

On the day of the study, a 25 mg/ml solution of propofol (MP Biomedicals, Solon, OH) in intralipid (20% emulsion) was freshly prepared. In all of the experiments, the mice were exposed to either propofol (MP Biomedicals, Solon, OH) 250 mg/kg or an equivalent volume of intralipid (vehicle control for propofol, Sigma RBI, St. Louis, MO) via intraperitoneal (i.p.) injection. The control mice were returned to their home cage at room temperature after injection. All of the anesthetized mice were initially anesthetized in their home cage, and, once sedated, they were transferred to a Thermocare® ICS therapy warmer unit (Thermocare, Incline Village, NV) either set to maintain a mouse body temperature of ∼37°C or with the heating element turned off, the latter setting used to achieve propofol-induced hypothermia. Rectal temperature was monitored using an electronic thermometer probe (Thermalert TH-5, Physitemp, Clifton, NJ).

### Preparation of Mouse Brain Protein Extracts

Following propofol or intralipid exposure, the mice were killed by cervical dislocation either 30 min, 2 h, or 6 h following drug or vehicle administration, then the brain was immediately removed and dissected in ice-cold Tris-EDTA buffer (Tris HCl 100 mM, 1 mM EDTA, pH 7.4). The hippocampal and cortical tissues were immediately frozen in liquid nitrogen and stored at −80°C. Protein extracts were prepared by mechanically homogenizing the hippocampal and cortical tissues in 5x vol/w RIPA buffer containing 50 mM Tris-HCl (Sigma-Aldrich, St. Louis, MO), pH 7.4, 1 mM ethylenediaminetetraacetic acid (EDTA), 150 mM NaCl, 0.1% SDS (Sigma), 0.5% sodium deoxycholate, 1% NP-40 (Sigma), phosphatase inhibitors (Cocktail 1 and 2, Sigma, 1∶100 dilution), and protease inhibitors (Cocktail set III, Calbiochem, San Diego, CA, 1∶200 dilution). The samples were incubated on ice for 30 min, sonicated for 30 s in pulse mode, and then centrifuged at 11,000 g for 10 min at 4°C. Total protein content was determined in the supernatant using the bicinchoninate (BCA) method [38].

### Cell Culture Studies and Cell Lysate Protein Extract Preparation

Propofol-induced tau phosphorylation was also examined using SH-SY5Y human neuroblastoma cells stably transfected to constitutively express human tau with 3 microtubule binding domains [39], [40], [41]. These Tau-SH-SY5Y cells were a kind gift of Dr. Luc Buée (Unité 422, INSERM, Lille, France). The cells were grown initially in 25-cm^2^ flasks containing Dulbecco's modified Eagle's medium (DMEM) with 10% fetal calf serum, 3 mM L-glutamine, 1 mM nonessential amino acids as well as 50 units/ml penicillin/streptomycin (DMEM and cell culture additives were purchased from Invitrogen, Carlsbad, CA), and were maintained in a 5% humidified incubator at 37°C. In preparation for anesthetic or vehicle exposure, the cells were transferred into 6-well plates and used at approximately 70% confluency.

On the day of the experiment, the growth medium was removed by aspiration, and each well was washed 2 times with 1 ml of fresh DMEM containing no additives. Immediately following the wash, each well received either 1.5 ml of Propofol (3 µg/ml) in 10% intralipid in DMEM or 10% intralipid in DMEM (vehicle). The concentration of propofol utilized was prepared using the commercial formulation of propofol (Diprivan®, AstraZeneca Pharmaceuticals, Wimington, DE) and based on a concentration previously utilized in cell culture studies [42]. This 3 µg/ml concentration of propofol (16.8 µM) is within the concentration range of that measured in human plasma following the clinical administration of this anesthetic [29]. The 6-well plates were incubated with propofol or vehicle for 1 h at 37°C. Following the 1 h exposure, the propofol or vehicle solutions were removed by aspiration, and each well was rinsed 2 times with 1 ml of ice-cold phosphate buffered saline. Furthermore, to confirm that the Tau-SH-SY5Y cells responded appropriately to treatments known to alter the tau phosphorylation state, we performed an additional set of experiments in these cells in which we incubated the cells for 1 h in normothermic (37°C) or hypothermic (30°C) conditions, the latter condition being known to increase tau phosphorylation [15], [30]. At the end of each treatment period, the cells were harvested by scraping in 200 µL of ice-cold RIPA buffer containing phosphatase- (1∶100, Sigma-Aldrich, St. Louis, MO) and protease (1∶200, EMD Chemicals, Gibbstown, NJ) inhibitors, and the samples were stored at −80°C until they were utilized in the immunoblotting analyses.

In preparation for SDS-PAGE and Western blot analysis, the cells were lysed on ice for 30 min and then sonicated on ice for 20 s in pulse mode at 30% power output. The lysate was then centrifuged at 1000 g for 10 min at 4°C, and the supernatant collected and analyzed for protein content using the bicinchoninate (BCA) method [38].

### SDS-PAGE and Western Blot Analysis

The expression of phosphorylated tau, total tau as well as the tau kinases and phosphatases was determined using SDS-polyacrylamide gel electrophoresis (PAGE) coupled with Western blot analysis. Brain homogenate or cell lysate aliquots containing 30 µg protein were separated on a SDS-10% polyacrylamide gel and then transferred onto nitrocellulose membranes (Amersham Biosciences, Pittsburgh, PA). Non-specific binding sites were blocked with 5% nonfat dry milk in Tris-buffered saline containing 0.1% Tween 20 for 1 h at room temperature and then were incubated overnight at 4°C with antibodies directed against either total tau, phosphorylated tau, a specific tau kinase or phosphatase. The following day the membranes were washed 3 times and then incubated for 1 h at room temperature with a horseradish peroxidase-linked secondary anti-mouse antibody (Cell Signaling Technology, Boston, MA, 1∶2500 dilution), and the immunoreactive band signal intensity was visualized by enhanced chemiluminescence (ECL Plus, GE Healthcare Biosciences, Piscataway, NJ). The immunoreactive bands were scanned using Fujifilm LAS 4000 imaging system (Fujifilm USA, Valhalla, NY) and densitometric analysis was performed on these scans with Image Gauge® analysis software (Fujifilm USA, Valhalla, NY).

### Detection of Total and Phosphorylated Tau

Antibodies were utilized in the study that were directed at tau phosphorylated at the following epitopes: AT8 (pSer^202^/pThr^205^ [43], 1∶250 dilution, Pierce Biotechnology), PHF-1 (pSer^396^/Ser^404^ [44], 1∶1000), MC6 (pSer^235^ [45], 1∶1000), CP13 (pSer^202^ [46], 1∶1000), pS262 (pSer^262^, 1∶1000; Invitrogen, Camarillo, CA, USA), and pS422 (pSer^422^; 1∶1000; Invitrogen). Total tau was detected using one of the following antibodies that detects all 6 six isoforms of tau: Tau46 (monoclonal, 1∶2500, Cell Signaling Technology, Danvers, MA), TG5 (monoclonal [47], 1∶1000), or Tau A0024 (polyclonal, 1∶10,000, Dako Cytomation). The PHF-1, CP13, MC6, and TG5 antibodies were a generous gift from Dr. Peter Davies (Albert Einstein University, New York, NY).

### Detection of Kinases and Phosphatases

Changes in tau kinases were examined using GSK-3β (1∶1000, BD Transduction Lab, Franklin Lakes, NJ), and the following antibodies purchased from New England Biolabs, Pickering, Ontario, Canada: phospho-GSK-3β (Ser^9^, 1∶1000), SAPK/JNK (1∶1000,), phospho-SAPK-JNK (Thr^183^/Tyr^185^, 1∶1000,), p44/42 MAPK (Erk 1/2, 1∶1000), phospho p44/42 MAPK (Erk 1/2, Thr^202^/Tyr^204^, 1∶1000). Changes in tau phosphatases were examined using PP2A-C subunit (Cell Signaling, 1∶1000), pan-calcineurin A (PP2B, Cell Signaling, 1∶1000), PP1 catalytic subunit (E-9, Santa Cruz, 1∶1000).

PP2A activity was assayed with the PP2A Immunoprecipitation Phosphatase BioAssay Kit from US Biological (Swampscott, MA, USA), according to the manufacturer instructions, and as described previously by us [48]. Briefly, brain hemispheres were homogenized in 40 x volume/weight of 20 mM imidazole-HCl pH 7.0, 2 mM EDTA, 2 mM EGTA, 1 mM PMSF, 10 µl/ml of Protease Inhibitor Cocktail P8340 (Sigma-Aldrich), and centrifuged at 2000 g for 5 min at 4°C. PP2A catalytic subunit was immunoprecipitated from the supernatant with a monoclonal antibody and protein A agarose for 2 hours at 4°C. The activity of the immunoprecipitated PP2A was assessed by the release of phosphate from a chemically synthesized phosphopeptide over a period of 10 min at 30°C. The amount of phosphate released was measured by the absorbance of the molybdate-malachite green-phosphate complex at 630 nm.

### Statistical Analysis

Group comparisons of immunoblot relative band intensities or PP2A assay results were performed using a one-way analysis of variance (ANOVA) with Newman-Keuls Multiple Comparison *post hoc* test applied when appropriate or by means of an unpaired *t*-test. The data were tested for normality using the Kolmogrov-Smirnov test. Statistical calculations were performed using Prism 4® software (GraphPad Software, Inc., San Diego, CA), and all data are reported as mean ± SD with a value of *P<*0.05 considered statistically significant.

### Immunofluorescence

Tissue fixation was done according to the “cold Bouin's method” developed in our laboratory [30]. Briefly, animals were decapitated, the brain was quickly removed and immersed in ice-cold Bouin's solution (saturated picric acid, formalin, acetic acid at 15∶5∶1) for 24 hr and embedded in paraffin blocks. Eight to ten µm thick sections were processed for immunofluorescent analyses. Deparaffinized and hydrated sections were incubated in 7% normal goat serum, 0.2% triton, 1% BSA in PBS 0.1 M at RT for 1 hr. The specimens were incubated with the primary antibodies diluted in PBS 0.1M containing 0.04% triton overnight at 4°C. The following antibodies were used: AT8 (monoclonal, 1∶200 dilution, Pierce Biotechnology, Rockford, IL) and Tau A0024 (polyclonal, 1∶1000, Dako Cytomation, Carpinteria, CA, USA). Bound antibodies were visualized with Alexa Fluor 488 conjugated anti-mouse IgG (1∶500 dilution) or Alexa Fluor 568 conjugated anti-rabbit IgG (1∶1000 dilution) (Molecular Probes, Eugene, OR). Slices were mounted with a Vectashield Hard Set Mounting Medium containing DAPI (Vector Laboratories, Inc, Burlingame, CA). Immunolabeled tissues were observed under a Carl Zeiss Axio Imager M2 (Zeiss, Jena, Germany) microscope equipped with an Axiocam MRm (Zeiss, Jena, Germany) and the Axiovision Rel.4.8 software.
